# Supplementary material for: A UMLS-based spell checker for natural language processing in vaccine safety
Source: BMC Med Inform Decis Mak. 2007 Feb 12;7:3. doi: 10.1186/1472-6947-7-3 (PMC1805499; doi:10.1186/1472-6947-7-3)
Supplement: Additional file 13 — RAP application directory. Sets up directory for RAP (RDF application for PHP) source code [file 1472-6947-7-3-S13.gz › rap/doc/phpdoc/ontModel/Individual.html]

xml version="1.0" encoding="iso-8859-1"?


Docs For Class Individual


## Class Individual

Description

Description |
Methods (details)

Interface that encapsulates an individual in an ontology, sometimes referred to as a fact or assertion.

In order to be recognised as an individual, rather than a generic resource, at least one rdf:type statement, referring to a known class, must be present in the model.

History: <LI>10-05-2004 : First version of this class.</LI>

- access: public
- author: Daniel Westphal <at d-westphal dot de mail at d-westphal dot de>
- version: V0.9.1

Located in /ontModel/Individual.php (line 29)

```
Object
   |
   --Node
      |
      --Resource
         |
         --ResResource
            |
            --OntResource
               |
               --Individual
```

Method Summary

Description |
Methods (details)

Individual
Individual
([string $uri = null])

Methods

Description |
Methods (details)

Constructor Individual (line 39)

Constructor You can supply a uri

- access: public

Individual

Individual
([string $uri = null])

- string
  $uri

#### Inherited Methods


Inherited From OntResource

> OntResource::OntResource()  
> OntResource::addComment()  
> OntResource::addIsDefinedBy()  
> OntResource::addLabelProperty()  
> OntResource::addProperty()  
> OntResource::addRDFType()  
> OntResource::addSeeAlso()  
> OntResource::asClass()  
> OntResource::asIndividual()  
> OntResource::asOntProperty()  
> OntResource::getComment()  
> OntResource::getInstanceRdfType()  
> OntResource::getIsDefinedBy()  
> OntResource::getLabelProperty()  
> OntResource::getPropertyValue()  
> OntResource::getRDFType()  
> OntResource::getSeeAlso()  
> OntResource::getVocabulary()  
> OntResource::hasComment()  
> OntResource::hasLabelProperty()  
> OntResource::hasRDFType()  
> OntResource::hasSeeAlso()  
> OntResource::isDefinedBy()  
> OntResource::listComments()  
> OntResource::listIsDefinedBy()  
> OntResource::listLabelProperties()  
> OntResource::listProperty()  
> OntResource::listRDFTypes()  
> OntResource::listSeeAlso()  
> OntResource::removeComment()  
> OntResource::removeDefinedBy()  
> OntResource::removeLabelProperty()  
> OntResource::removeProperty()  
> OntResource::removeRDFType()  
> OntResource::removeSeeAlso()  
> OntResource::setComment()  
> OntResource::setInstanceRdfType()  
> OntResource::setIsDefinedBy()  
> OntResource::setLabelProperty()  
> OntResource::setPropertyValue()  
> OntResource::setRDFType()  
> OntResource::setSeeAlso()  
> OntResource::setVocabulary()

Inherited From ResResource

> ResResource::ResResource()  
> ResResource::addProperty()  
> ResResource::equals()  
> ResResource::getAssociatedModel()  
> ResResource::getIsAnon()  
> ResResource::getProperty()  
> ResResource::hasProperty()  
> ResResource::listProperties()  
> ResResource::removeAll()  
> ResResource::removeProperties()  
> ResResource::setAssociatedModel()  
> ResResource::setIsAnon()  
> ResResource::setURI()

Inherited From Resource

> Resource::Resource()  
> Resource::equals()  
> Resource::getLabel()  
> Resource::getLocalName()  
> Resource::getNamespace()  
> Resource::getURI()  
> Resource::toString()

Inherited From Object

> Object::toString()

Documentation generated on Thu, 7 Jul 2005 13:41:28 +0200 by phpDocumentor 1.3.0RC3
